# OLID I: an open leaf image dataset for plant stress recognition

**DOI:** 10.3389/fpls.2023.1251888

**Published:** 2023-09-12

**Authors:** Nabil Anan Orka, M. Nazim Uddin, Fardeen Md. Toushique, M. Shahadath Hossain

**Affiliations:** ^1^ Department of Electrical and Electronic Engineering, Islamic University of Technology (IUT), Gazipur, Bangladesh; ^2^ Olericulture Division, Horticulture Research Center (HRC), Bangladesh Agricultural Research Institute (BARI), Gazipur, Bangladesh; ^3^ Entomology Section , Horticulture Research Center (HRC), Bangladesh Agricultural Research Institute (BARI), Gazipur, Bangladesh

**Keywords:** plant stress recognition, multi-label classification, crop nutritional deficiency, automated pest management, precision agriculture, computer vision, machine vision

## Introduction

Plants undergo stress whenever they are subjected to adverse conditions or an element that inhibits metabolism and growth (Lichtenthaler, 1996). Plants incur irreversible harm, even death, when overtaxed under unfavorable circumstances for an extended period (Pahlich, 1993). Plant stress is caused by two types of environmental conditions: biotic stressors, or living creatures such as fungus, bacteria, and insects, and abiotic stressors, or non-living elements such as drought, salinity, and a dearth of minerals (Mosa et al., 2017). Plant stressors drastically impair agricultural productivity. Crop-wise yield losses aggravated by these detrimental organisms can be severe, ranging from 26.3% to 40.3% (Oerke, 2006). Based on a study (Gatehouse et al., 1992), an estimated 37% of global agricultural yield is lost due to pests and pathogens, while 13% is lost due to insects. In addition, crop nutritional deficiencies endanger over two billion people’s food security (Gaikwad et al., 2020), reducing crop yield by up to 70% (Francini and Sebastiani, 2019). Precision agriculture strives to address these issues by facilitating the use of better resources and continuously enhancing the food supply’s sustainability. It has been extensively demonstrated that precision agriculture is an indispensable ingredient of streamlined pest management and nutrition monitoring systems (Mavridou et al., 2019; Nugroho et al., 2020; Presti et al., 2022).

Artificial intelligence (AI) continues to strengthen its influence in various fields because of its constant innovations and utilization of robust applications to solve complex problems that conventional computer systems and human beings cannot successfully handle. The growing acceptance of AI is not an exception to precision agriculture. In fact, data-driven AI applications contribute significantly to the discipline (Linaza et al., 2021). Machine vision systems, for example, have a widespread application in the control of herbicides, livestock, and crops (Dhanya et al., 2022). For AI to learn and enhance accuracy over time, an abundance of readily accessible data is imperative. However, in precision agriculture, the effort and costs associated with data collection and annotation, as well as laboratory analysis, make dataset preparation painstaking (Lu and Young, 2020). On the contrary, open access data alleviates such complications. As a result, it stimulates new projects and ensures reproducible outcomes.

The PlantVillage dataset (Hughes et al., 2015) continues to be the mainstay of computer vision tasks associated with plant stress identification since its debut. By far, the largest public dataset of leaf images is the PlantVillage dataset, which consists of 54,309 healthy and unhealthy leaf images divided into 38 categories by species and diseases. The efforts linked with tomatoes (*Solanum lycopersicum*) can help comprehend the scope of PlantVillage. Because the collection contains the most tomato pictures, a plethora of research is devoted to the identification of tomato pests and pathogens (Nandhini and Ashokkumar, 2021; Tan et al., 2021; Al-gaashani et al., 2022; Bhujel et al., 2022). TensorFlow, Python’s open-source machine learning framework, features two plant disease datasets, PlantaeK (Kour and Arora, 2022) and PlantLeaves (Chouhan et al., 2019). The PlantDoc dataset contains internet-curated images of 17 diseases across 13 plant species (Singh et al., 2020). There are also some crop-specific open access archives for dealing with rice (*Oryza sativa*) (Raksarikon, 2021) and sugar beet (*Beta vulgaris*) (Yi et al., 2020) nutritional deficiencies. However, notable research gaps continue to persist.

In practice, a single leaf could exhibit several irregularities (McCauley et al., 2009). However, no agricultural dataset encompasses multiple labels or categories in a single shot. Aside from tomatoes, the PlantVillage dataset contains two other vegetables, but all of the documented crops and plants are widely cultivated in the United States. According to an FAO assessment, around 6000 plant species are produced for food, with 200 species offering considerable food quantities worldwide (Bélanger et al., 2019). As a result, there remains innumerable crop anomalies that must be addressed. In the PlantaeK and PlantLeaves datasets, the complete set of pictures is divided into only two broad categories: healthy and diseased. The issue with the PlantDoc dataset is that plants can be stressed without displaying visible indications (McCauley et al., 2009). As such, it raises some questions about the reliability of the labeling. Finally, there is no public dataset containing images of both abiotic and biotic stresses over an extensive spectrum of crops.

With the purpose of a freely accessible, expert annotated collection of healthy, nutritiously depleted and pest-impaired leaf photos, we propose a dataset encompassing the principal crops in Bangladesh, namely tomato (*Solanum lycopersicum*), eggplant (*Solanum melongena*), cucumber (*Cucumis sativus*), bitter gourd (*Momordica charantia*), snake gourd (*Trichosanthes cucumerina*), ridge gourd (*Luffa acutangula*), ash gourd (*Benincasa hispida*), and bottle gourd (*Lagenaria siceraria*). The dataset comprises 4,749 high-resolution (3024 x 3024) images organized into 57 distinct categories. In addition to strengthening insect and disease management for the aforementioned crops, the collection aims to fill the data shortage in the field of crop nutrition deficiency.

The main contributions of our dataset include:

- The largest number of classes featured in an agro-domain dataset.
- The first multi-label classification challenge in agriculture.
- The inaugural open-access dataset to cover symptoms of both biotic and abiotic stressors across multiple crops at the same time, establishing a benchmark in plant stress recognition.

## Data collection and labeling

From March 17, 2022 to May 5, 2023, we used an iPhone 13 Pro Max to capture 5000 leaf samples from observational fields in Bangladesh Agricultural Research Institute (BARI), Gazipur. We solely employed the primary 12 MP wide camera with an f/1.5-aperture lens. Apart from the exposure setting, which was set at -1, we did not modify any default parameters when capturing the photos. This was determined because when the images were overexposed to sunshine, the computational software system altered the shots in such a way that the leaves differed radically from what they seemed to naked eyes. The images were captured directly overhead with a 1:1 aspect ratio, leading to a resolution of 3024 x 3024. The flash was switched off.

We opted to collect samples in natural lighting environments rather than controlled ones to ensure generalizability of the algorithms trained on the dataset. We set up our data collecting equipment — a table and a camera mounted on a tripod — right adjacent to fields with plenty of natural light. During the course of a day’s data collection, we monitored illuminances with a Digital LX1330B Illuminance Meter. We avoided bias by, first, sampling at random with no specific plant stress in mind, and second, maintaining identical heights from the table’s base to the phone camera for each crop. The exact spacing between the camera and the leaf/table are listed below:

- Ash gourd - 22.5 cm
- Bitter gourd - 14 cm
- Bottle gourd - 27.5 cm
- Cucumber - 24 cm
- Eggplant - 23 cm
- Ridge gourd - 23 cm
- Snake gourd - 22 cm
- Tomato - 15 cm

Instead of photographing leaves in plants with a complex background that included several leaves, soil, and other plant elements, we sampled one leaf at a time and placed it on top of a satin fabric with a homogeneous black tone. In our dataset, we encountered the early stages of crop anomalies, when the differentiating traits are difficult to spot visually. Such complexities mandated a consistent background with just one leaf in succession, rendering the distinct features more obvious. In addition, the PlantVillage dataset (Hughes et al., 2015), widely regarded as the gold standard in automated plant disease identification, relied on a grey or black paper sheet as a background. As a result, we used an analogous method whilst employing the same black satin cloth for each snapshot. Furthermore, we observed that the black backdrop accentuated the colors of the symptoms while ensuring that no shadows were formed to introduce scene complexity. Finally, researchers frequently implement segmentation to extract the leaves from complicated surroundings (Luo et al., 2023). Fortunately, because of the uniform black backdrop, segmentation is not necessary; hence, we ensured ease of use.

Following the acquisition, the samples were handed over to a laboratory, where an experienced team meticulously analyzed and labeled each image. The team performed both perceptual labeling and laboratory analytical labeling. Here, each leaf was visually identified first, followed by rigorous laboratory examination. Nevertheless, we did not impose any specific criterion in order to allow the team to carry out the annotation procedure to the best of their scientific knowledge while minimizing bias. We adopted this dual approach for two reasons. First of all, plants are susceptible to stress irrespective of whether they exhibit any clear visual cues (McCauley et al., 2009). Second of all, sun rays reaching the leaves might occasionally mimic the appearance of several symptoms, even when the plants were not truly affected (Barbedo, 2019). The chemical analysis, for these reasons, was indispensable for dealing with the challenges.

The annotation was overseen by two researchers (M.N.U. and M.S.H.) with over 15 years of expertise in the subject. In the event of a disagreement amongst team members, the supervisors (M.N.U. and M.S.H.) served as mediators, using their professional judgments.

## Data description

**Figure 1:**
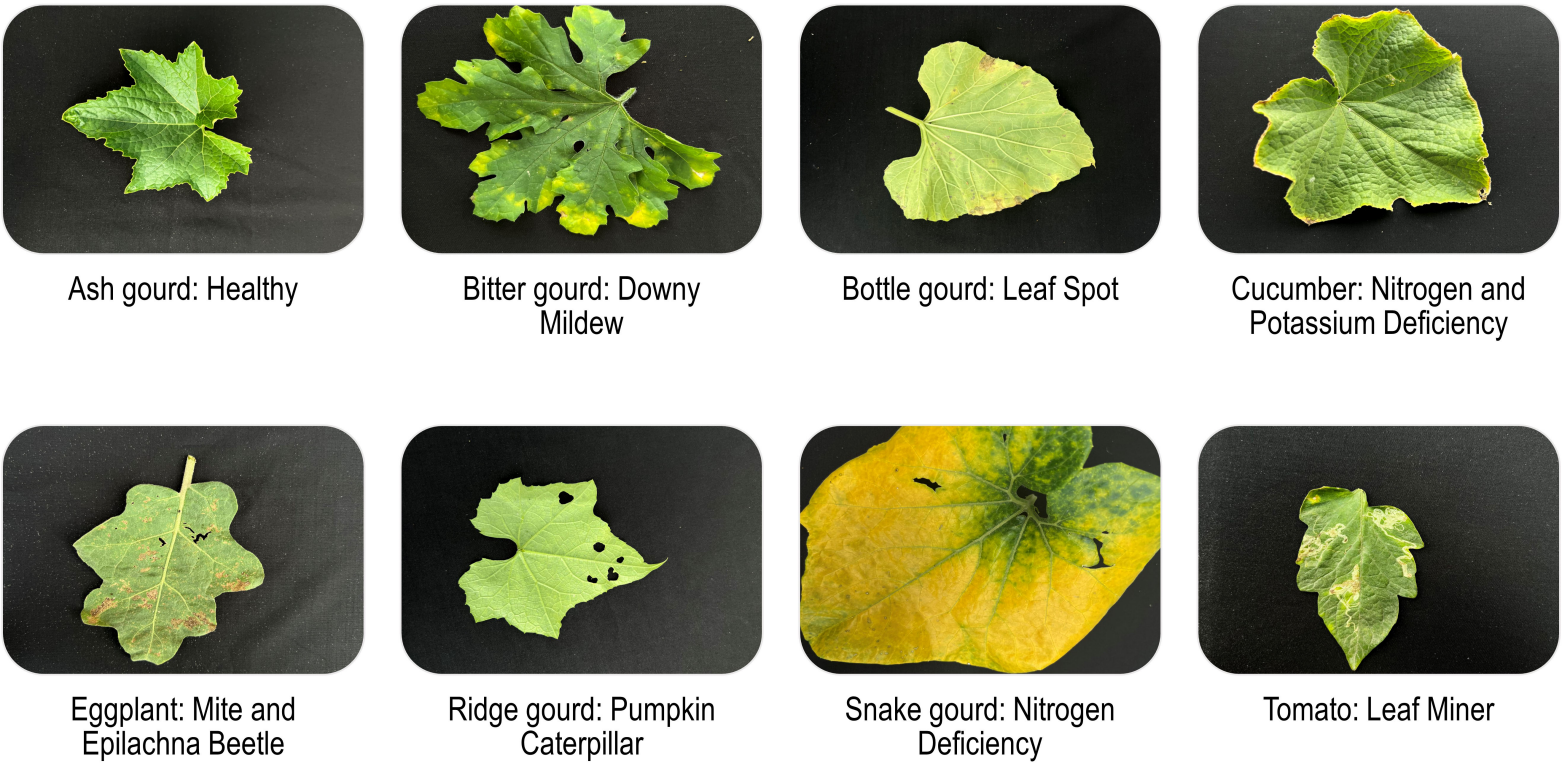
Example members of the OLID I dataset.

Despite acquiring 5000 photos categorized into 110 classes, we opted not to include some of the classes due to a significant imbalance in class representation. For example, powdery mildew on bottle gourd and eggplant aphid had just one sample apiece. The number of phosphorus deficiency representatives of different crops was insufficient as well. As such, we omitted classes with fewer than 10 samples to avoid skewed predictive accuracies towards the majority classes. The final dataset comprises 4,749 images grouped into 57 distinct classes. **Table 1** describes a thorough overview of the dataset. It should be emphasized that the dataset is labeled and designed for the classification of the secondary classes listed in **Table 1**. We provided broad abiotic and biotic categories as primary classes so that researchers could easily comprehend the dataset and, if required, customize it for their specific goals and applications. In addition, we incorporated both the dorsal and ventral surfaces since symptoms are frequently found on either side. **Figure 1** depicts example representatives of a few of the 57 classes. In our dataset, most insect categories, such as beetles and mites, show the signs or symptoms of pest-infestation. However, in several instances, the insects themselves were noticeable.

**Table 1:** Class distribution of the OLID I dataset.

Crop	Primary Class	Secondary Class Abbrv.	Secondary Class Full Form	Sample Size
Ash gourd	Healthy			83
	Disease	PM	Powdery Mildew	79
	Nutritional Deficiency	K	Potassium Deficiency	293
		K Mg	Potassium and Magnesium Deficiency	53
		N	Nitrogen Deficiency	61
		N K	Nitrogen and Potassium Deficiency	386
		N Mg	Nitrogen and Magnesium Deficiency	42
Bitter gourd	Healthy			181
	Disease	DM	Downy Mildew	48
		LS	Leaf Spot	35
	Insect	JAS	Jassid	35
	Nutritional Deficiency	K	Potassium Deficiency	55
		K Mg	Potassium and Magnesium Deficiency	40
		N	Nitrogen Deficiency	147
		N K	Nitrogen and Potassium Deficiency	128
		N Mg	Nitrogen and Magnesium Deficiency	116
Bottle gourd	Healthy			31
	Disease	DM	Downy Mildew	28
		LS	Leaf Spot	28
	Insect	JAS	Jassid	24
		JAS MIT	Jassid and Mite	29
	Nutritional Deficiency	K	Potassium Deficiency	30
		N	Nitrogen Deficiency	39
		N K	Nitrogen and Potassium Deficiency	102
		N Mg	Nitrogen and Magnesium Deficiency	34
Cucumber	Healthy			34
	Nutritional Deficiency	K	Potassium Deficiency	50
		N	Nitrogen Deficiency	89
		N K	Nitrogen and Potassium Deficiency	76
Eggplant	Healthy			92
	Insect	EB	Epilachna Beetle	74
		FB	Flea Beetle	36
		JAS	Jassid	34
		MIT	Mite	75
		MIT EB	Mite and Epilachna Beetle	95
	Nutritional Deficiency	K	Potassium Deficiency	106
		N	Nitrogen Deficiency	67
		N K	Nitrogen and Potassium Deficiency	106
Ridge gourd	Healthy			70
	Insect	PLEI	Pumpkin Leaf Eating Insect	80
		PLEI IEM	Pumpkin Leaf Eating Insect and Insect Egg Mass	40
		PLEI MIT	Pumpkin Leaf Eating Insect and Mite	25
		PC	Pumpkin Caterpillar	33
	Nutritional Deficiency	N	Nitrogen Deficiency	152
		N Mg	Nitrogen and Magnesium Deficiency	34
Snake gourd	Healthy			59
	Disease	LS	Leaf Spot	33
	Nutritional Deficiency	K	Potassium Deficiency	56
		N	Nitrogen Deficiency	102
		N K	Nitrogen and Potassium Deficiency	206
Tomato	Healthy			236
	Insect	LM	Leaf Miner	207
		MIT	Mite	200
		JAS MIT	Jassid and Mite	32
	Nutritional Deficiency	K	Potassium Deficiency	36
		N	Nitrogen Deficiency	47
		N K	Nitrogen and Potassium Deficiency	40

### Multi-label classification

In the agri-sector, our dataset is a pioneer in multi-label classification, where multiple categories appear in a single snapshot. We provided two leaves with multiple stress symptoms to demonstrate. **Figure 1** exhibits a cucumber leaf with a pale green tint in the center and yellow tips, indicating nitrogen and potassium shortages, respectively. Similarly, the eggplant leaf, which includes numerous pits of varied sizes as well as yellow and brown speckles cluttered all over, demonstrates both beetle and mite infestations. **Table 1** intends to assist researchers in identifying leaves that have multiple labels or classes. For example, ‘N Mg’ denotes an absence of nitrogen and magnesium, while ‘JAS MIT’ represents leaves afflicted with both jassids and mites. The underscore refers to the existence of multiple categories in an image, which were diligently grouped and organized within the dataset in adequately named folders.

### A contrast with PlantVillage

The primary objective of our dataset is to be utilized for plant stress recognition. The PlantVillage dataset has consistently served as the benchmark in this context. Nevertheless, researchers recently achieved 100% accuracy on it (Bruno et al., 2022). We aspired to offer a successor to PlantVillage with additional stressor categories encompassing hitherto unexplored crops and high-resolution photographs.

The presence of multiple labels in individual photos, which the PlantVillage collection lacks, is perhaps the most valuable aspect of OLID I. Another aspect that separates our dataset from PlantVillage is that our dataset encompasses 57 classes to PlantVillage’s 38, including 16 multi-label classes. Finally, despite the gourd family’s impact in our global nutritional needs (Rolnik and Olas, 2020), there is still a significant lack in research on cucurbits stress detection, which we seek to fulfill.

## Data usage notes

OLID I is available on Kaggle (Orka et al., 2023a) and Zenodo (Orka et al., 2023b). In Zenodo, we uploaded the dataset in sections so that individuals with limited network access can view it more easily. In addition, we supplied an excel file with complete breakdown of the classes in both databases.

The dataset is fairly straightforward for setting up because we put photographs in folders that correspond to the proper annotations. Segmentation will not be required since we settled on persistent background. However, as the dataset is imbalanced, we advocate any form of augmentation before training different algorithms. For example, the Augmentor package (Bloice et al., 2017) in Python has horizontal flip, 90-degree rotation, vertical flip, random rotation, random shear, random skew, and random zoom functions that can be used to increase sample sizes for particular classes and balance the dataset without compromising variance. In addition, generative AI could potentially be utilized to create highly lifelike samples (Lu et al., 2022). We acknowledge that, due to the scarcity of high-performance equipment, many researchers will be unable to fully use the high-resolution photographs; nevertheless, cloud-based solutions, such as Google Colab, can effortlessly overcome this limitation. Moreover, the images can be readily scaled to accommodate resource restrictions. Finally, while our dataset comprises Red-Green-Blue (RGB) photographs, color transformation is an alternative that has previously shown promising results (Krishnaswamy Rangarajan and Purushothaman, 2020; Schuler et al., 2021).

## Closing remarks

We believe our dataset will encourage researchers to embark on novel endeavors that will stretch their abilities. In particular, the dataset will inspire more realistic detection algorithms that can recognize many stressors in a single picture. With a plethora of plant species and stress categories, OLID I offers a dataset that embraces scientific rigor and aims to eliminate oversights in data labeling, reducing the likelihood of feeding erroneous data to the algorithm and creating misinterpretation. The effective use of our dataset will result in considerable improvements in plant stress recognition, while simultaneously building trust in AI.

## Data availability statement

The datasets presented in this study can be found in online repositories. The names of the repository/repositories and accession number(s) can be found below: Zenodo (https://zenodo.org/record/8105154) and Kaggle (https://www.kaggle.com/datasets/raiaone/olid-i).

## Author contributions

NO: Conceptualization, Methodology, Investigation, Visualization, Writing - Original Draft; MU: Methodology, Validation, Resources, Data Curation, Supervision; FT: Investigation, Resources, Writing - Review and Editing; MH: Validation, Data Curation, Supervision. All authors contributed to the article and approved the submitted version.
